# Photo-fermentative bacteria aggregation triggered by L-cysteine during hydrogen production

**DOI:** 10.1186/1754-6834-6-64

**Published:** 2013-05-03

**Authors:** Guo-Jun Xie, Bing-Feng Liu, De-Feng Xing, Jun Nan, Jie Ding, Nan-Qi Ren

**Affiliations:** 1 State Key Laboratory of Urban Water Resource and Environment, Harbin Institute of Technology, 73 Huanghe Road, P.O. Box 2614, Harbin, 150090, China

**Keywords:** Bioflocculation, Photo-hydrogen production, L-Cysteine, Extracellular polymeric substances, Disulfide bonds, DLVO

## Abstract

**Background:**

Hydrogen recovered from organic wastes and solar energy by photo-fermentative bacteria (PFB) has been suggested as a promising bioenergy strategy. However, the use of PFB for hydrogen production generally suffers from a serious biomass washout from photobioreactor, due to poor flocculation of PFB. In the continuous operation, PFB cells cannot be efficiently separated from supernatant and rush out with effluent from reactor continuously, which increased the effluent turbidity, meanwhile led to increases in pollutants. Moreover, to replenish the biomass washout, substrate was continuously utilized for cell growth rather than hydrogen production. Consequently, the poor flocculability not only deteriorated the effluent quality, but also decreased the potential yield of hydrogen from substrate. Therefore, enhancing the flocculability of PFB is urgent necessary to further develop photo-fermentative process.

**Results:**

Here, we demonstrated that L-cysteine could improve hydrogen production of *Rhodopseudomonas faecalis* RLD-53, and more importantly, simultaneously trigger remarkable aggregation of PFB. Experiments showed that L-cysteine greatly promoted the production of extracellular polymeric substances, especially secretion of protein containing more disulfide bonds, and help for enhancement stability of floc of PFB. Through formation of disulfide bonds, L-cysteine not only promoted production of EPS, in particular the secretion of protein, but also stabilized the final confirmation of protein in EPS. In addition, the cell surface elements and functional groups, especially surface charged groups, have also been changed by L-cysteine. Consequently, absolute zeta potential reached a minimum value at 1.0 g/l of L-cysteine, which obviously decreased electrostatic repulsion interaction energy based on DLVO theory. Total interaction energy barrier decreased from 389.77 KT at 0.0 g/l of L-cysteine to 127.21 kT at 1.0 g/l.

**Conclusions:**

Thus, the strain RLD-53 overcame the total energy barrier and flocculated effectively. After a short settlement, the biomass rush out will be significantly reduced and the effluent quality will be greatly improved in the continuous operation. Furthermore, aggregation of PFB could enable high biomass hold-up of photobioreactor, which allows the photobioreactor to operate at low hydraulic retention time and high organic loading rate. Therefore, the described flocculation behaviour during photo-hydrogen production is potentially suitable for practicable application.

## Background

Global growing concerns about energy shortages and the environmental pollution have led to worldwide use of renewable energy. Hydrogen is considered as a viable energy carrier for the future which could play an important role in the reduction of emissions of greenhouse gases [1,2]. Recently, biological hydrogen production processes, especially photo-fermentative hydrogen production by PFB has been attracting more and more attention, as it utilizes various renewable sources like biomass and sunlight to produce an ideal, renewable and carbon-free energy for the future [3]. However, it should be realized that most of photo-fermentative processes are based on suspended culture [4-6] in which it is difficult to achieve high biomass concentration, effective retention and separation of PFB biomass, resulting from poor flocculation of PFB [7]. For steady-state operation of photobioreactor, due to the poor flocculability, PFB cells cannot be efficiently separated from supernatant and rush out with effluent from reactor continuously. This increased the effluent turbidity, meanwhile led to increases in pollutants like chemical oxygen demand, total nitrogen, and total phosphate, causing poor effluent water quality. Furthermore, to replenish the biomass washout, substrate was continuously utilized for cell growth rather than hydrogen production [8,9]. Thus, the poor flocculability not only deteriorated the effluent quality, but also decreased the potential yield of hydrogen from substrate. Therefore, enhancing the flocculability of PFB is urgent necessary to further develop photo-fermentative process.

Previous studies also tried to isolate self-flocculated PFB or enhance flocculation of PFB, but successful case was rare. Watanabe [10] first and only reported that photosynthetic bacteria *Rhodovulum sp*. PS88 has a self-flocculating activity. And high density cell culture was obtained under continuous cultivation in a single-tower fermenter [11]. However, there was no report about photo-hydrogen production and flocculation mechanism of *Rhodovulum sp*. PS88. According to the DLVO theory, the PFB, *Rhodopseudomonas acidophila*, could not overcome the total energy barrier to flocculate effectively, because contribution of van der Waals interaction energy to the total interaction energy could be neglected resulting from the small effective Hamaker constant (2.27×10^-23^ J) [12]. As a result, *R. acidophila* could not overcome the total energy barrier to flocculate effectively. So far, the information about PFB could flocculate and simultaneously improve hydrogen production have been not yet reported, and effective method and detailed mechanism of flocculation in photo-fermentation hydrogen production is still lacking.

In this work, we first time found that the L-cysteine induced the obvious bioflocculation of *Rhodopseudomonas faecalis* RLD-53 and at the same time promoted hydrogen production. Traditionally, flocculability of biological cells highly depended on the extracellular polymeric substances (EPS) [13], bacterial surface characteristics [14] and electrolyte concentration [15]. However, L-cysteine is unique natural amino acids containing a thiol group, which could form disulfide bond. Disulfide bonds are crucial to the folding and stability of many proteins [16,17], usually proteins secreted to the extracellular medium. As a predominant component in EPS, proteins have been demonstrated to play a crucial role in the bacterial aggregation [18,19]. Therefore, the mechanism of aggregation triggered by L-cysteine was explored through combination biological function of L-cysteine and traditional flocculation theory. EPS, surface properties and zeta potential of PFB were investigated for better understanding flocculation characteristics of strain RLD-53 under different concentration of L-cysteine. Effect of disulfide bonds on components of EPS production and conformational changes of proteins in EPS were also determined. Furthermore, contribution of specific EPS protein conformation and cell surface functional groups to bacterial aggregation were further discussed. Finally, the DLVO theory was used to evaluate the flocculability of *R. faecalis* RLD-53.

## Results and discussion

### Hydrogen production and bioflocculation of *R. faecalis* RLD-53

**Figure 1:**
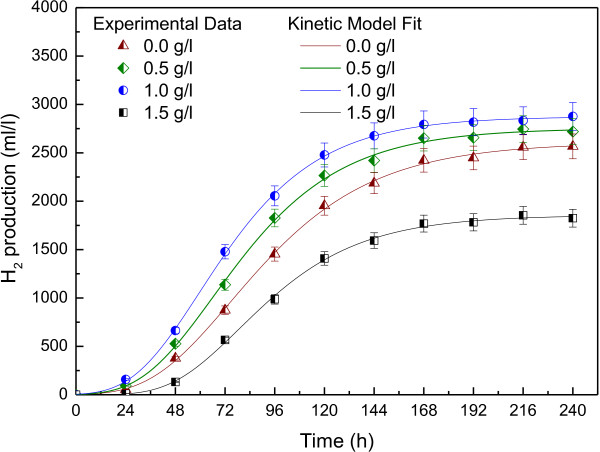
**Hydrogen production kinetics of *R. faecalis *RLD-53 at different L-cysteine concentrations.**

Hydrogen productions were carried out at different L-cysteine concentrations (0.5, 1.0 and 1.5 g/l) in batch culture and the control was no addition of L-cysteine. After cumulative hydrogen production was obtained, modified Gompertz equation was used as kinetic model to determine the hydrogen production kinetics of *R. faecalis* RLD-53 at different L-cysteine concentrations (Figure 1). The hydrogen production kinetic parameters at different L-cysteine concentrations were shown in Table 1. The maximum cumulative hydrogen production (*H*_*max*_) and maximum production rate (*R*_*max*_) increased with increasing L-cysteine concentration from 0.0 to 1.0 g/l, but decreased with further increasing the L-cysteine concentrations from 1.0 to 1.5 g l/l. At 1.0 g/l of L-cysteine, hydrogen production started rapidly with the lag phase of about 27.46 h, reaching the maximum hydrogen yield of 2.58 mol H_2_/mol acetate and production rate of 32.85 ml/l/h, respectively. However, at 1.5 g/l of L-cysteine, hydrogen production started slowly, did not reach the maximum rate of 20.81 ml/l/h until lag time 46.10 h and hydrogen yield was only 1.66 mol H_2_/mol acetate. The results indicated that a proper concentration of L-cysteine (1.0 g/l) could promote the hydrogen production, while excessive L-cysteine depressed the hydrogen productivity.

**Table 1:** Effect of L-cysteine concentration on hydrogen production kinetics and nitrogenase activity

**L-cysteine (g/l)**	**H**_**2 **_**yield (mol H**_**2**_**/mol acetate)**	***H***_***max ***_**(ml/l)**	***R***_***max ***_**(ml/l/h)**	***λ *(h)**	***r***^***2***^	**Nitrogenase activity (nmol C**_**2**_**H**_**4**_**/ml/h)**
0.0	2.33±0.106	2610±118	24.97	36.22	0.998	957±85
0.5	2.46±0.123	2755±137	29.35	31.77	0.997	1125±78
1.0	2.58±0.129	2878±144	32.85	27.46	0.994	1374±94
1.5	1.66±0.114	1859±127	20.81	46.10	0.996	765±69

In order to further demonstrate above results, acetylene reduction was used to determine the activity of nitrogenase, which catalysed the hydrogen production in photofermentation [3]. As shown in Table 1, nitrogenase activities increased with the L-cysteine concentration, reached maximum (1374 nmol C_2_H_4_/ml/h) at 1.0 g/l. However, with further increase of L-cysteine to 1.5 g/l, the nitrogenase activity sharply decreased to765 C_2_H_4_/ml/h. Thiol group from L-cysteine is a key part of active sites in nitrogenase, which play an important role in structure and function of nitrogenase [20,21]. In chemical evolution of a nitrogenase model, the ratio of thiol and molybdenum significantly influenced the catalytic activity, and the maximum catalytic activity was obtained at ratio of 1:1 [22]. In this study, nitrogenase activity was enhanced by increasing of thiol from L-cysteine, but strongly depressed by excessive L-cysteine.

**Figure 2:**
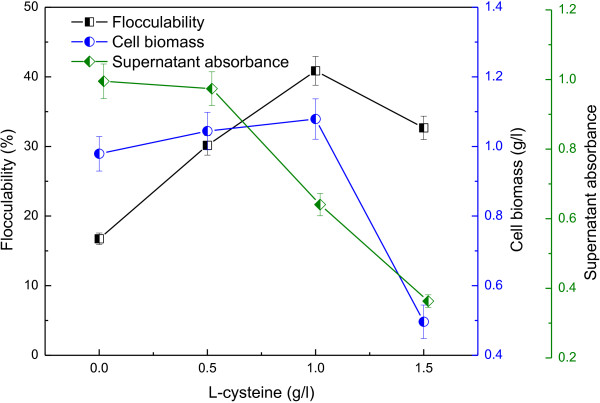
**Cell growth and flocculability of *R. faecalis *RLD-53 at different L-cysteine concentrations.**

Figure 2 showed the cell growth and flocculability of *R. faecalis* RLD-53 at various L-cysteine concentrations. Cell biomass increased with the concentration of L-cysteine from 0 to 1.0 g/l, reached maximum (1.08 g/l) at 1.0 g/l, and then decreased sharply with further increase of L-cysteine to 1.5 g/l. The results suggested that excessive L-cysteine depressed cell growth of RLD-53. In the previous study, L-cysteine was also found to inhibit the growth of *Neurospora crassa* at high concentration, due to itself rather than its metabolism products [23]. The flocculability increased from 16.73 to 40.86% with an increase in L-cysteine concentration from 0.0 to 1.0 g/l, and then decreased. Above results indicated that suitable concentration of L-cysteine could significantly enhance the flocculability of *R. faecalis* RLD-53 and also promote photo-hydrogen production. After standing for 5 minutes, the absorbance of the supernatant decreased with increasing L-cysteine concentration. This suggested that after a short settlement, the biomass rush out could be significantly reduced and the effluent quality could be greatly improved in the continuous operation. Therefore, the described flocculation behaviour of *R. faecalis* RLD-53 during photo-hydrogen production is potentially suitable for practicable application.

**Figure 3:**
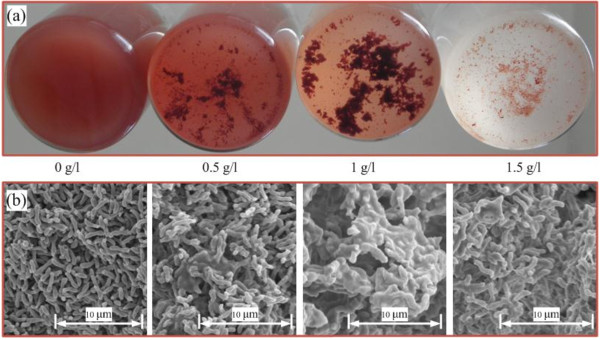
**Bioflocculation of *R. faecalis *RLD-53 at different concentration of L-cysteine.** (**a**) photo of bioflocculation; (**b**) SEM images of bioflocculation.

As seen from Figure 3a, bioflocculation formation increased with the concentration of L-cysteine, and best floc was observed at 1.0 g/l. And then, bioflocculation decreased with L-cysteine, due to weak cell growth at high concentration of L-cysteine. The result showed that L-cysteine could cause the remarkable flocculation of *R. faecalis* RLD-53. After formation (about 48 hours), the floc was stable in whole hydrogen production, which indicated that flocculation of *R. faecalis* RLD-53 could be applied in the continuous hydrogen production. Scanning electron microscope (SEM) analysis also showed that there were clear morphological differences of bioflocculation at different concentration of L-cysteine (Figure 3b). Loosely structure was observed in the control (0 g/l of L-cysteine) and there were almost not formation of floc. Interaction adhesion among cells increased and formed a stable structure through covered and tightly linked together by EPS at 1.0 g/l of L-cysteine. The amount of EPS on the cell surface was increased with the L-cysteine from 0.0 to 1.0 g/l, indicating that proper concentration of L-cysteine may promote the production of EPS. The porous structure of the floc was also observed at 1.0 g/l. Such structure was likely to facilitate the passage of substrate and the release of hydrogen gas as well as light penetration.

### EPS of *R. faecalis* RLD-53

**Figure 4:**
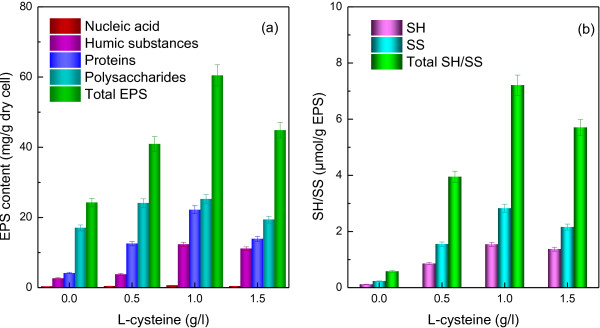
**EPS compositions of *R. faecalis *RLD-53 at various concentration of L-cysteine.** (**a**), EPS component; (**b**), thiol group (SH) and disulfide bond (SS) content in EPS.

EPS have been reported as a major component in microbial aggregates and play a crucial role in bioflocculation formation [24]. Characteristics and the contents of the EPS have significant effect on the formations and properties of microbial aggregates [13]. Figure 4 illustrated the effect of L-cysteine concentrations on EPS chemical compositions. The total EPS content increased from 24.23 to 60.47 mg/g dry cell with the increase of L-cysteine from 0 to 1.0 g/l. A further increase in L-cysteine concentrations decreased of EPS content, due to inhibition from excessive L-cysteine. The polysaccharides, proteins and humic substances were the major component of EPS from strain RLD-53. Compared with the control (0 g/l), the EPS at 1.0 g/l of L-cysteine increased by 36.24 mg/g dry cell, mainly (18.03 mg/g) from increase of protein secretion. Previous studies reported that with high content of negatively charged amino acids, protein is more interacted than polysaccharides with multivalent cations for stabilizing floc structure [18]. Removal of proteins from the sludge floc resulted in deflocculation, which demonstrated the important function of protein in formation of floc [19]. These results indicated that L-cysteine promoted the production of extracellular polymeric substances, especially protein secretion, which facilitated bioflocculation formation.

In addition, humic substances also increased significantly (Figure 4). Humic substances are a natural organic matter, resulting from the biodegradation of dead biomass, which are resistant to degradation [25]. In the pure culture of photofermentative bacteria, humic substances mainly came from the dead cell decomposition. In free cell culture, cell debris and humic substances from the dead cell decomposition may disperse into the culture broth. However, after the bioflocculation formation, humic substances from dead cell decomposition may be retained in the EPS matrix. As a result, the humic substances also significantly increase, due to the floc formation caused by L-cysteine.

**Figure 5:**
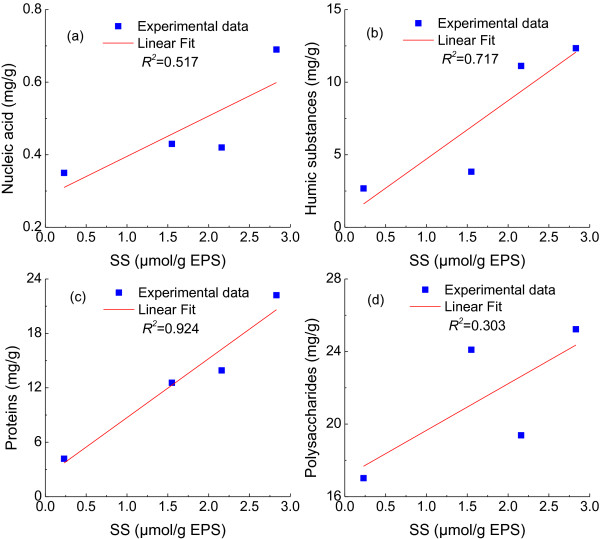
**Relationship between disulfide bonds and components of EPS production.** (**a**), Nucleic acid; (**b**), Humic substances; (**c**), Proteins; (**d**), Polysaccharides.

Disulfide bonds formation from L-cysteine are crucial to the folding and stability of many proteins [16,17], usually proteins secreted to the extracellular medium. Figure 4b presented disulfide bonds concentrations in the EPS at different L-cysteine concentration. The disulfide bonds content in EPS increased with the increase of L-cysteine concentration, and reached maximum at 1.0 g/l of L-cysteine (Figure 4b), corresponding to the above mentioned maximum proteins production in EPS (Figure 4a). Disulfide bonds detected in the control may come from the inoculation containing a small amount of L-cysteine. The relationships between disulfide bonds and components of EPS production were also investigated, as shown in Figure 5. Compared with other components of EPS, proteins had the most significant positive linear relationship with disulfide bonds based on correlation coefficients (*R*^*2*^=0.924) (Figure 5). In the previous studies, bovine pancreatic trypsin inhibitor (a model protein to investigate protein structure and folding pathways) secretion efficiency was significantly decreased by disulfide removal [26], while proteins secretion in *Pichia pastoris* were enhanced by overexpression of protein disulfide isomerase, which helped in rearrangement of incorrect disulfide pairings [27]. Therefore, our results implied that the formation of disulfide bonds from L-cysteine stimulated the secretions of proteins and it is beneficial to stabilize floc.

**Figure 6:**
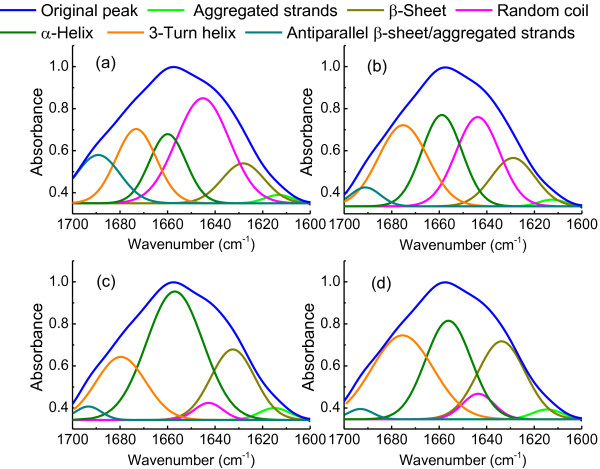
**Spectral decomposition of the amide I bands at different concentration of L-cysteine (g/l).** (**a**), 0.0; (**b**), 0.5; (**c**), 1.0; (**d**), 1.5.

In addition, the biological function of disulfide bonds includes stabilization of protein structure as well as determining the pathway and efficiency of protein folding [28,29]. Thus, the potential impacts of L-cysteine on the conformations of EPS proteins were further explored based on amide I region (1600–1700 cm^-1^) in the Fourier Transform Infrared Spectroscopy (FTIR) spectra [30] (Figure 6). The percentages of secondary structure were determined from the areas of the individual assigned bands and their fraction of the total area in the amide I (Table 2). In the control (0 g/l), random coil was predominant in EPS proteins, while 3-Turn helix, α-Helix, β-Sheet and antiparallel β-sheet/aggregated strands also had considerable proportion. With the increasing of L-cysteine, the content of random coil and antiparallel β-sheet/aggregated strands significantly decreased, while α-Helix and β-Sheet increased. These results indicated that the protein conformations were greatly changed by disulfide bonds from L-cysteine.

**Table 2:** **Conformation changes of EPS proteins from *R. faecalis *RLD-53 at different concentration of L-cysteine**

**Secondary structures**	**Wavenumber (cm**^**-1**^**)**	**L-cysteine (g/l)**			
		**0.0**	**0.5**	**1.0**	**1.5**
Aggregated strands (%)	1625-1610	1.55	1.21	2.51	2.01
β-Sheet (%)	1640-1630	11.33	14.73	21.09	25.31
Random coil (%)	1645-1640	37.50	27.30	3.28	5.52
α-Helix (%)	1657-1648	16.96	25.52	49.40	31.03
3-Turn helix (%)	1666-1659	20.13	27.63	21.35	34.54
Antiparallel β-sheet/ aggregated strands (%)	1680-1695	12.53	3.62	2.38	1.59

**Figure 7:**
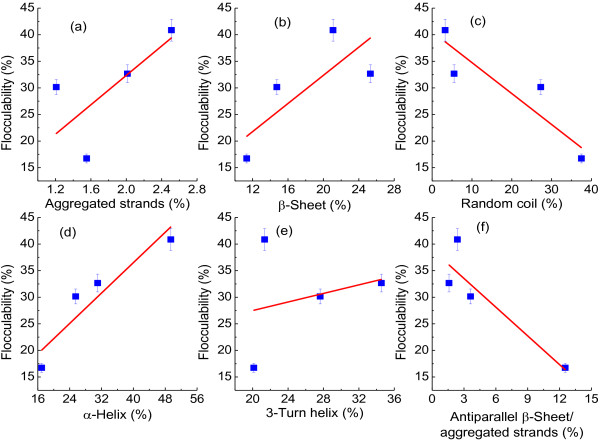
**Contribution of EPS protein conformations to flocculability of *R. faecalis *RLD-53.** (**a**), Aggregated strands; (**b**), β-Sheet; (**c**), Random coil; (**d**), α-Helix; (**e**), 3-Turn helix; (**f**), Antiparallel β-sheet/aggregated strands.

Conformation of protein could be influenced by contact time with the matrix [31], hydrophobicity of surface [32] and curvature of matrix [33]. Such changes of protein molecules on bacterial surface were not reversible. So, the conformational changes of proteins were suggested as driving force for bacterial adhesion to solid matrix [34]. In this study, the contribution of specific conformation of EPS protein to flocculability of PFB were further analysed in Figure 7. Aggregated strands, β-sheets and α-helices promoted bioflocculation formation (Figure 6a, b and d), but random coils and antiparallel β-sheet/aggregated strands deteriorated flocculability (Figure 6c and f). However, there was no obvious corresponding relationship between 3-Turn helix and flocculability (Figure 6e).

### Cell surface functional groups of *R. faecalis* RLD-53

With increasing protein and EPS covering on the cell surface, the surface elemental composition and functional groups could be greatly influenced by L-cysteine. The surface elements and functional groups of *R. faecalis* RLD-53 were studied by X-ray photoelectron spectroscopy (XPS) (Additional file 1: Figure S1), which detected the outermost molecular layers (mainly EPS) of the cell surface (2–5 nm). The major peaks in the spectra identified by XPS were the C 1s and O 1s, and N 1s peak, with minor peaks of P, Na, Cl and Si. The functional groups on the cell surfaces were illustrated by high-resolution XPS spectra of the C 1s, O 1s and N 1s region in Additional file 2: Figure S2. The C 1s spectra were resolved into four individual component groups: C_G1_, 284.6 ev, C-(C, H) mainly from hydrocarbons; C_G2_, 286.2 eV, C-(O, N) from proteins and alcohols; C_G3_, 287.8 eV, C=O or O-C-O from carboxylate, carbonyl, amide, acetals, or hemiacetals, and C_G4_, 289.2 eV, O=C-OH and O=C-OR commonly from uronic acids. The O 1s peak was decomposed into two peaks, O_G1_, 531.3 eV, O=C from carboxylate, carbonyl, ester, or amide, and O_G2_, 532.7 eV, O-(C, H) from hydroxide, acetal, and hemiacetal. The N 1s peak was also resolved into two component peaks, N_G1_, 399.9 eV, O=C-NH-R from amines and amides, and N_G2_, 401.3 eV, C-NH_2_ mainly from basic amino acids. The percentages of surface functional groups were determined from the XPS peaks area after subtraction of a linear background (Table 2). The results showed that the functional groups on the cell surface of *R. faecalis* RLD-53 were significantly affected by L-cysteine concentration.

**Figure 8:**
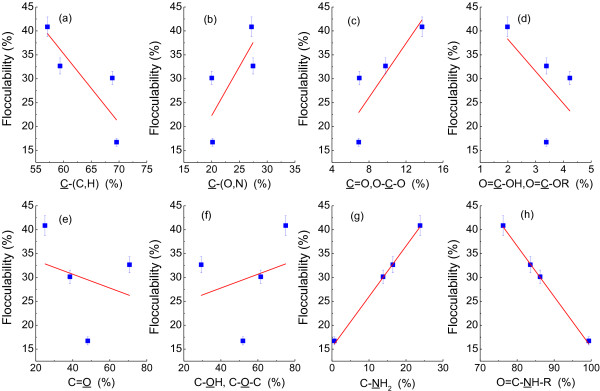
**Contribution of cell surface functional groups to flocculability of *R. faecalis *RLD-53.** (**a**), C-(C, H); (**b**), C-(O, N); (**c**), C=O, O-C-O; (**d**), O=C-OH, O=C-OR; (**e**), C=O; (**f**), C-OH, C-O-C; (**g**), C-NH_2_; (**h**), O=C-NH-R.

The contribution of cell surface functional groups to flocculability of PFB was presented in Figure 8. Result showed that the present of C-(O, N) and C=O or O-C-O on the cell surface promoted photo-fermentative bacteria flocculation, while compounds with C-(C, H) and O=C-OH, O=C-OR on the cell surface deteriorated the flocculability (Figure 8a-d). The flocculability of *R. faecalis* RLD-53 and content of O-(C, H) followed the same trends changed with concentration of L-cysteine, but no significant linear correlation (Figure 8e and f). In addition, the C-NH_2_ on the cell surface promoted bioflocculation whereas the O=C-NH-R decreased bioflocculation ability (Figure 8g and h). Previously, some reports suggested that O=C-OH groups play an important role in flocculation [35], but others emphasized the importance of C-NH_2_[36]. In this work, the presence of C-NH_2_ promoted microorganism flocculation whereas O=C-OH, O=C-OR hampered bioflocculation (Figure 8e and f). Therefore, the change of cell surface functional groups caused by L-cysteine was conducive to flocculation of PFB.

### Zeta potential of *R. faecalis* RLD-53

**Figure 9:**
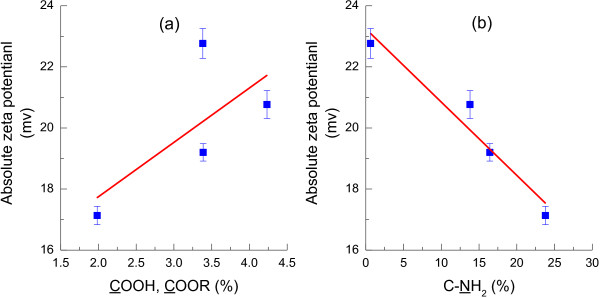
**Effect of charged groups on the zeta potential of *R. faecalis *RLD-53.** (**a**), COOH, COOR; (**b**), C-NH_2_.

Zeta potential is also an important parameter which characterizes the physicochemical properties of the bacterial cell envelope and plays an important role in aggregation and disaggregation processes [37]. The absolute zeta potential decreased with the L-cysteine concentration, and reached minimum at 1.0 g/l (Additional file 3: Figure S3). This was mainly attributable to the changes in cell surface characteristics, especially changes in the functional groups caused by L-cysteine. Bacterial surface charge mainly originated from ionization of surface groups [38], including negatively charged (e.g., carboxyl, phosphoryl or sulfhydryl groups of carbohydrates and proteins) and positively charged groups (amine groups of amino acids, amino sugars) [37]. In this study, cell surface of *R. faecalis* RLD-53 mainly contained COOH and C-NH_2_ as charged groups (Table 3). With increasing of COOH, COOR content, the absolute zeta potential increased, due to the negative contribution to cell surface charge from ionization of COOH group (Figure 9). On the contrary, absolute zeta potential decreased with the content of C-NH_2_ group, because negative net surface charge decreased by positive charge from ionization of NH_2_ group.

**Table 3:** Results of the high-resolution XPS analysis of the C 1s, O 1s and N 1s peak region from cell surface

**L-cysteine (g/l)**	**C 1s (%)**				**O 1s (%)**		**N 1s (%)**	
	**C**_**G1 **_**284.8 eV**	**C**_**G2 **_**286.2 eV**	**C**_**G3 **_**287.8 eV**	**C**_**G4 **_**289.2 eV**	**O**_**G1 **_**531.3 eV**	**O**_**G2 **_**532.7 eV**	**N**_**G1 **_**399.1 eV**	**N**_**G2 **_**400.05 eV**
	**C-(C,H)**	**C-(O, N)**	**C=O, O-C-O**	**O=C-OH, O=C-OR**	**C=O**	**C-OH, C-O-C**	**O=C-NH-R**	**C-NH**_**2**_
0.0	69.55	20.17	6.89	3.38	48.09	51.91	99.36	0.64
0.5	68.79	20.03	6.95	4.23	38.47	61.53	86.19	13.81
1.0	57.10	27.18	13.73	1.98	24.98	75.02	76.20	23.80
1.5	59.38	27.45	9.78	3.39	70.64	29.36	83.57	16.43

### Flocculability evaluated by DLVO theory

DLVO theory has been widely applied as both qualitative and quantitative models to explain microbial adhesion and aggregation [39,40]. Here, DLVO theory was applied to predict the potential energy barrier that hindered aggregation of *R. faecalis* RLD-53 at different concentration of L-cysteine.

**Figure 10:**
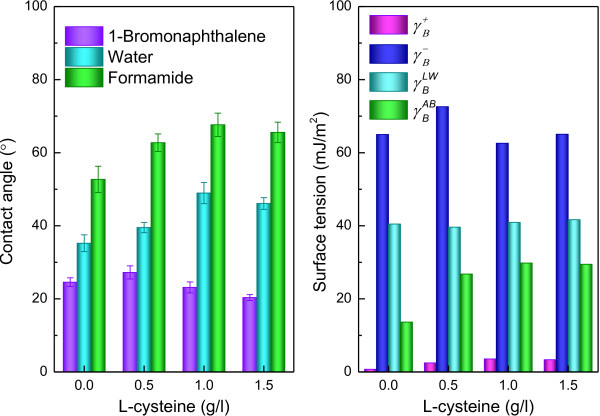
**Contact angle and surface thermodynamic properties of *R. faecalis *RLD-53.** (**a**), Contact angle; (**b**), surface thermodynamic properties.

Application of the DLVO approach requires the surface thermodynamic parameters. Surface thermodynamic properties of *R. faecalis* RLD-53 cell were calculated through the measurement of the contact angles with of three different liquids (water, formamide, and 1-bromonaphthalene) (Figure 10). Cell surface hydrophobicity was determined by water contact angle *θ*_*W*_. Traditionally, surfaces are divided into two categories: wetting (*θ*_*W*_ < 90°) and non-wetting (*θ*_*W*_ > 90°). It is worth noting that hydrophobic interactions between surfaces become effective at *θ*_*W*_ > 65° and hydrophilic interactions at *θ*_*W*_ < 65° [41]. In this study, the all of water contact angles (*θ*_*W*_ <65°) indicated that cell surface was hydrophilic and hydrophilic interactions were effective between cells of *R. faecalis* RLD-53. Hydrophilic interaction is the proclivity of strongly polar chains, molecules or particles for repelling each other in aqueous [41]. The hydrophilic interactions decreased with increasing the water contact angle resulting from different L-cysteine concentrations. This was favourable for the formation of biofloc.

**Figure 11:**
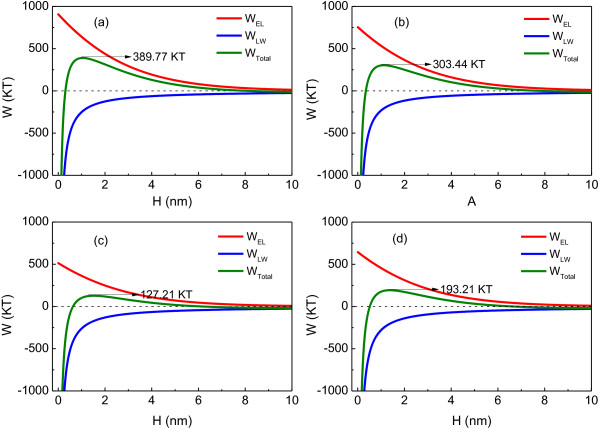
**Interaction energy profiles as a function of cells distance at various L-cysteine concentrations (g/l).** (**a**), 0.0; (**b**), 0.5; (**c**), 1.0; (**d**), 1.5.

The total interaction energies between the microbial cells were calculated as a function of the separation distance between the cell and cell in 0.01 mol/l NaCl (Figure 11). In the control, interaction energy barrier was 389.77 KT. The high energy barrier indicated stable cell suspension, corresponding to poor flocculability of *R. faecalis* RLD-53 at 0.0 g/l of L-cysteine (Figure 2). The energy barrier dropped from 389.77 to 127.21 kT with an increase in L-cysteine concentration to 1.0 g/l. Hence, the flocculability of *R. faecalis* RLD-53 increased with the increasing L-cysteine concentration (Figure 2). However, when the L-cysteine concentration was further increased to 1.5 g/l, bacterial cell growth and EPS production were depressed, resulting in increase of zeta potential and electrostatic repulsion (*W*_*EL*_). Consequently, the interaction energy barrier rebounded to 193.21 KT. Therefore, the flocculability of *R. faecalis* RLD-53 decreased at 1.5 g/l of L- cysteine.

The poor flocculability of photosynthetic H_2_-producing bacterium, *R. acidophila*, was attributed to its inherent surface characteristics [12]. The effective Hamaker constant between *R. acidophila* and water was only 2.27×10^-23^ J, resulting in the negligible contribution of van der Waals interaction energy (*W*_*LW*_) to the total interaction energy. As a result, the interaction energy barrier between cells was up to 1665 KT in 0.01 mol/l NaCl solutions. Consequently, the bacterial cells could not overcome the total energy barrier to flocculate effectively. In this study, the effective Hamaker constant between *R. faecalis* RLD-53 and water was 5.54×10^-21^ J at 1.0 g/l of L-cysteine, suggesting that van der Waals interaction energy was important for total interaction energy. In addition, the electrostatic repulsive energy (*W*_*EL*_) decreased with decreasing of absolute zeta potential, resulting from changes of surface charged groups caused by L-cysteine. The interaction energy barrier between cells of *R. faecalis* RLD-53 was 127.21 KT. Therefore, *R. faecalis* RLD-53 flocculate effectively at 1.0 g/l of L-cysteine.

## Conclusions

In this work, L-cysteine was found to promote the effective flocculation and photo-hydrogen production of *R. faecalis* RLD-53. This finding suggested L-cysteine can be applied as flocculant for continuous photo-hydrogen production. Results showed that proper L-cysteine concentration (1g/l) improved flocculability and hydrogen productivity of *R. faecalis* RLD-53. The reasons of flocculation also were analysed. Through formation of disulfide bonds, L-cysteine not only promoted production of EPS, in particular the secretion of protein, but also stabilized the final confirmation of protein in EPS. Research also noted that the cell surface covered by EPS have been changed by L-cysteine, thus absolute zeta potential decreased with L-cysteine and reached minimum at 1.0 g/l, which greatly decreased electrostatic repulsion interaction energy based on DLVO theory. Further analysis indicated that total interaction energy barrier decreased from 389.77 KT at 0.0 g/l of L-cysteine to 127.21 kT at 1.0 g/l. This led to the *R. faecalis* RLD-53 overcome the total interaction energy barrier and flocculate effectively. Therefore, forming stable floc caused by L-cysteine offers great advantages for the realization of enhancing production yield and scale-up application in bio-hydrogen production.

A better understanding the flocculation behaviour of PFB triggered by L-cysteine not only could help the design of subsequent hydrogen production process by flocculation of PFB, but also might favour the further understanding of the bioflocculation mechanism.

## Methods

### Bacterium, medium and culture conditions

The photo-hydrogen producer used in this study was *Rhodopseudomonas faecalis* RLD-53 [42]. Acetate was used as the sole carbon source, and glutamate was used as nitrogen source in the medium for hydrogen production. The culture medium of strain RLD-53 was prepared as described in previous report [42].

The batch culture experiments were carried out in triplicate with 80 ml of the medium in 100 ml sealed reactors and filled with argon to maintain anaerobic conditions [43].The reactors were autoclaved at 121°C for 15 min. *R. faecalis* RLD-53 in the mid-exponential growth phase was inoculated into reactors. The light intensity on the outside surface of the reactors was maintained at 150 W/m^2^ by incandescent lamps (60 W). The reactors were stirred at 120 rpm at constant temperature of 35°C.

### Hydrogen production kinetics

Modified Gompertz equation has been widely accepted and used to determine the kinetic parameters of hydrogen production [44]. After cumulative hydrogen production curves were obtained over the course of an entire batch experiment, modified Gompertz equation was used to describe the hydrogen production kinetics at different L-cysteine concentrations:

(1) $$H=H_{\text{max}}\text{exp}\left\{-\text{exp}\left[\frac{R_{\text{max}}e}{H_{\text{max}}}\left(\lambda -t\right)+1\right]\right\}$$

Where *t* is culture time (h); *H* is cumulative H_2_ production (ml/l medium); *H*_*max*_ is maximum cumulative H_2_ production (ml/l medium); *e*=2.71828; *R*_*max*_ is maximum H_2_ production rate (ml/l/h); and λ is the lag-phase time (h).

### Scanning electron microscope (SEM)

Surface morphology of the bioflocculation samples was evaluated by a scanning electron microscope [45]. The bioflocculation samples were fixed with 2.5% glutaraldehyde and left for 1.5 h in a 4°C refrigerator. The samples were gently washed with phosphate buffer solution and then dehydrated by successive passages through 50%, 70%, 80%, 90%, and 100% ethanol. Each rinsing and dehydrating step took 10 min. The samples were refreeze dried (Hitachi E-2030, Japan) for 4 h, subsequently coated with gold powder by Sputter Coater (Hitachi E-1010, Japan) and finally attached on to the microscope supports with silver glue. Scanning electron microscope images were taken at 5 kV using an SEM (Hitachi S-3400N, Japan).

### X-ray photoelectron spectroscopy (XPS)

Surface elements concentrations and functional groups on cell surface were determined by the XPS method, which detected the outermost molecular layers (mainly EPS) of the cell surface (2–5 nm) [46]. After 72 h cultivation, *R. faecalis* RLD-53 cultured at different L-cysteine concentrations were harvested by centrifugation at 12000 rpm for 10 min and washed twice with double distilled water. Collected cell samples were placed in a freeze-dryer for about 48 h (until freeze-dried). XPS was carried out on a PHI-5600 equipped with a monochromatic Al Kα source and data acquisition and processing were conducted using the PC Access ESCA version 7.2A program. The anode voltage and power were 12.5 kV and 250 W, respectively. The pressure in analysis chamber was maintained at 10^-9^ Torre during each measurement. All binding energies were referenced to the C 1s neutral carbon peak at 284.6 eV. Spectra were analysed using XPSPeak software (Version 4.1).

### Zeta potential and flocculability tests

The bacterial cells cultured at different L-cysteine concentrations were harvested by centrifugation at 12000 rpm for 10 min and washed twice with 0.9 % NaCl solution. The bacterial cells were resuspended in 0.01 mol/l NaCl solution. These cell suspensions were used for the zeta potentials measurement (Nano-ZS, Malvern Co., UK) and flocculability tests. The absorbance of prepared cell suspensions was also measured with a spectrophotometer (Shimadzu UV-2550; Shimadzu, Kyoto, Japan) at 650 nm (*A*_*0*_). Thereafter, the cell suspensions were centrifuged at 1000 rpm for 2 min, and the supernatant optical density was measured again at 650 nm (*A*_*t*_). Thus, the flocculability of *R. faecalis* RLD-53 value can be calculated using the following equation.

(2) $$F\%=\left(1-\frac{A_{t}}{A_{0}}\right)\times 100\%$$

### EPS extraction

EPS was extracted using cation exchange resin [47] (Dowex Marathon C, 20–50 mesh, sodium form, Fluka 91973). The bacterial cells were collected by centrifugation at 12000 rpm for 10 min. And then the cells were washed twice with 0.9% NaCl solution. Subsequently, the cells were re-suspended in ddH_2_O and transferred to an extraction beaker. And then the beaker was added resin (70 g/g-VSS) and stirred at 600 rpm for 12 h at 4°C. The samples was centrifuged at 12000 g for 30 min followed by filtration using a 0.45 μm cellulose acetate membrane to remove resin, microorganisms, and residual debris to obtain an EPS sample for further analysis.?>

### Fourier transform infrared spectroscopy (FTIR)

The FTIR spectra of EPS samples were determined using a Fourier transform infrared spectrophotometer (Spectrum One-B, Perkin Elmer, U.S.). The freeze-dried EPS samples were ground with infrared grade KBr and press into pellets and used for FTIR measurement. For each sample, 350–400 scans were collected over the spectral range of 400–4000 cm^-1^ at a resolution of 4 cm^-1^. The protein conformation was analysed from the amide I region [30]. Component peaks were fitted with Gaussian band profiles using the frequencies of the components deduced from the second derivatives.

### Interaction energy evaluated by DLVO approach

Application of the DLVO approach required the surface thermodynamic parameters, which was determined by measuring the contact angles and using the Lifshitz van der Waals acid–base approach [48].

The DLVO interaction energy (*W*_*Total*_) between two bacterial cells can be calculated as the sum of the van der Waals (*W*_*LW*_) and electrostatic (*W*_*EL*_) interaction energies [49,50]:

(3) $$W_{\text{Total}}=W_{\text{LW}}+W_{\text{EL}}$$

where

(4) $$W_{\text{LW}}=-\frac{A_{\text{BLB}}R}{12H}$$

(5) $$W_{\text{EL}}=2\mathrm{\pi \epsilon R}\psi_{s}{}^{2}\ln\left(1+\text{exp}\left(-\mathrm{\kappa H}\right)\right)$$

Where *A*_*BLB*_ is the effective Hamaker constant. *H* is the separation distance between the cells. *R* is the cell radius of *R. faecalis* RLD-53, determined by the Malvern Mastersizer 2000 (Malvern Instruments Ltd., UK). *ψ*_*s*_ and κ represent the stern potential and inverse of the Debye length respectively, which are related to the electric double layer interaction *W*_*EL*_. *ψ*_*s*_ could be replaced by zeta potential measurement and *κ* can be calculated from different electrolyte concentrations.

### Analytical method

Light intensity was measured at the surface of reactor with solar power meter TENMARS TM-207 (Tenmars Electronics CO., LTD., Taiwan, China). Biogas was sampled from the head space of the photobioreactor by using gas-tight glass syringes and hydrogen content was determined by using a gas chromatograph (Agilent 4890D, Agilent Technologies, USA). The gas chromatograph column was Alltech Molesieve 5A 80/100. Argon was used as the carrier gas with a flow rate of 30 ml/min. Temperatures of the oven, injection, detector, and filament were 35°C, 120°C, 120°C, 140°C, respectively. Residual acetate in culture broth was determined using a second gas chromatograph (Agilent 7890 A, Agilent Technologies, USA) equipped with a flame ionization detector. The liquor samples were firstly centrifuged at 12,000 rpm for 5 min, and filtered through a 0.2 μm membrane before free acids were analyzed. The operational temperatures of the injection port, the column and the detector were 220, 190 and 220°C, respectively. Nitrogen was used as carrier gas at flow rate of 50 ml/min.

Whole-cell nitrogenase activity was assayed by acetylene reduction following the procedure in our previous report [51]. The polysaccharide content in EPS was determined by the anthrone method [52] using glucose as a standard. The protein and humic substance in EPS were measured followed the modified Lowry method [53] using bovine serum albumin and humic acid (Fluka Chemical Corp., USA) as the respective standards. The nucleic acid content was measured by the diphenylamine colorimetric method [54] using fish DNA as the standard. Thiol (SH) and disulfide bond (SS) in EPS were determined using 5-5′-dithio-bis (2-nitrobenzoic acid) (DTNB) according to the method of Ellman [37] and the procedure reported by Kalapathy et al. [55].

## Abbreviations

PFB: Photo-fermentative bacteria; DLVO: Derjaguin–Landau–Verwey–Overbeek; EPS: Extracellular polymeric substances; FTIR: Fourier Transform Infrared Spectroscopy; SEM: Scanning electron microscope; XPS: X-ray photoelectron spectroscopy; DTNB: 5-5^′^-dithio-bis (2-nitrobenzoic acid).

## Competing interests

The authors declare that they have no competing interests.

## Authors’ contributions

All authors contributed intellectually via scientific discussions during the work and have read and approved the final manuscript. Guo-Jun Xie designed the study, executed the experimental work, data interpretation and drafted the manuscript. Bing-Feng Liu participated in experimental design and data interpretation, and reviewed the manuscript. De-Feng Xing helped the determination of nitrogenase activity. Nan Jun made a Hypothesis to explain humic substances increase. Ding Jie commented on the manuscript and contributed to the design of the study. Nan-Qi Ren contributed to the design of the study, data interpretation and reviewed the manuscript.

## Supplementary Material

Additional file 1: Figure S1 XPS spectra of *R. faecalis* RLD-53 at different concentration of L-cysteine (g/l). (a), 0.0; (b), 0.5; (c), 1.0; (d), 1.5. Click here for file

Additional file 2: Figure S2 High-resolution fitted C 1s, O 1s and N 1s spectra of *R. faecalis* RLD-53 at different concentration of L-cysteine. Click here for file

Additional file 3: Figure S3 Absolute zeta potential of *R. faecalis* RLD-53 at different concentration of L-cysteine. Click here for file
