# Supplementary figures and images for: Photo-fermentative bacteria aggregation triggered by L-cysteine during hydrogen production
Source: Biotechnol Biofuels. 2013 May 3;6:64. doi: 10.1186/1754-6834-6-64 (PMC3648407; doi:10.1186/1754-6834-6-64)

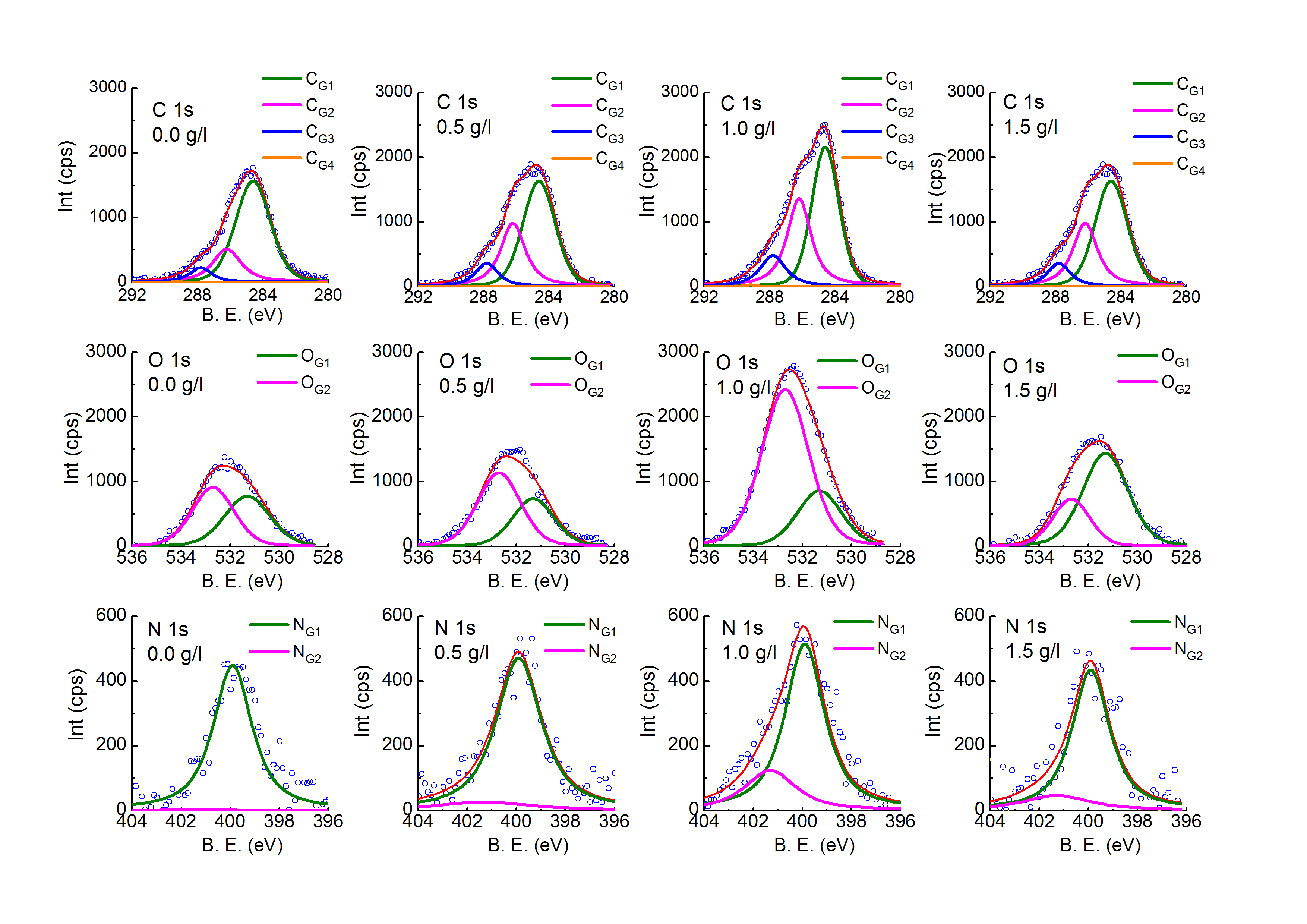


Figure S2

Supplement: Additional file 2: Figure S2 — High-resolution fitted C 1s, O 1s and N 1s spectra of R. faecalis RLD-53 at different concentration of L-cysteine. [file 1754-6834-6-64-S2.doc]
